# Pre-treatment loss to follow-up in adults with pulmonary TB in Kenya

**DOI:** 10.5588/pha.23.0059

**Published:** 2024-03-01

**Authors:** M. N. Mulaku, E. Ochodo, T. Young, K. R. Steingart

**Affiliations:** ^1^ Centre for Global Health Research, Kenya Medical Research Institute, Kisumu, Kenya;; ^2^ Centre for Evidence-based Health Care, Division of Epidemiology and Biostatistics, Faculty of Medicine and Health Sciences, Stellenbosch University, Tygerberg, South Africa;; ^3^ Department of Pharmacology, Clinical Pharmacy, and Pharmacy Practice, Faculty of Health Sciences, University of Nairobi, Nairobi, Kenya;; ^4^ Department of Clinical Sciences, Liverpool School of Tropical Medicine, Liverpool, UK

**Keywords:** bacteriologically confirmed TB, PTLFU, PTB, treatment initiation, patient losses

## Abstract

**SETTING:**

County referral hospital in western Kenya.

**OBJECTIVES:**

To ascertain the proportion of pre-treatment loss to follow-up (PTLFU) and associated patient factors in adults with pulmonary TB (PTB) in western Kenya.

**DESIGN:**

A retrospective data review of laboratory and treatment registers for adults with bacteriologically confirmed PTB between January 2018 to December 2021. We defined PTLFU as failure to initiate treatment within 14 days of diagnosis. We used multivariable logistic regression modelling to identify patient factors associated with PTLFU.

**RESULTS:**

Of 476 patients with PTB, 67.2% were male; the mean age was 36.1 years; 37.0% were HIV-positive; 5.7% had a history of anti-TB treatment; 40.6% were not traceable in the treatment register; 202 (42.4%, 95% CI 38.1–46.9) experienced PTLFU. Age ≥55 years (aOR 2.6, 95% CI 1.0–6.7) and providing only an address (aOR 34.2, 95% CI 18.7–62.5) or only a telephone contact number (aOR 22.3, 95% CI 3.5–141.1) were associated with PTLFU. Sex, HIV status, history of anti-TB treatment and place of residence were not associated with PTLFU.

**CONCLUSION:**

PTLFU contributes markedly to TB patient losses in western Kenya. Strengthening systems for documenting patient information and actively monitoring PTLFU are crucial for attrition reduction.

## Abstract (fr)

**CONTEXTE:**

Hôpital de référence du comté dans l'ouest du Kenya.

**OBJECTIFS:**

Vérifier la proportion de perte de suivi avant traitement (PTLFU) et les facteurs de patients associés chez les adultes atteints de TB pulmonaire (PTB) dans l'ouest du Kenya.

**DESIGN:**

Un examen rétrospectif des données des registres de laboratoire et de traitement pour les adultes atteints de PTB confirmée bactériologiquement entre janvier 2018 et décembre 2021. Nous avons défini le PTLFU comme l'incapacité à initier un traitement dans les 14 jours suivant le diagnostic. Nous avons utilisé un modèle de régression logistique multivariable pour identifier les facteurs des patients associés au PTLFU.

**RÉSULTATS:**

Sur 476 patients atteints de PTB, 67,2% étaient des hommes ; l'âge moyen était de 36,1 ans ; 37,0% étaient séropositifs ; 5,7% avaient des antécédents de traitement antituberculeux ; 40,6% n'étaient pas traçables dans le registre des traitements ; 202 (42,4% ; IC 95% 38,1–46,9) ont subi un PTLFU. L'âge ≥55 ans (aOR 2,6 ; 95% CI 1,0–6,7) et le fait de ne fournir qu'une adresse (aOR 34,2 ; 95% CI 18,7–62,5) ou qu'un numéro de téléphone (aOR 22,3 ; 95% CI 3,5–141,1) étaient associés à la PTLFU. Le sexe, le statut VIH, les antécédents de traitement antituberculeux et le lieu de résidence n'étaient pas associés à la PTLFU.

**CONCLUSION:**

La PTLFU contribue de manière significative aux pertes de patients tuberculeux dans l'ouest du Kenya. Le renforcement des systèmes de documentation des informations sur les patients et le suivi actif de la PTLFU sont essentiels pour réduire l'attrition.

Kenya has a high TB burden with an incidence rate of 251/100,000 population and mortality rates of 40 and 21/100,000 population for HIV-negative and HIV-positive people, respectively (2021).^1^ The country was one of the few to meet the WHO End TB 2020 targets.^1,2^ Nonetheless, Kenya is currently classified by the WHO as a high-burden country for TB and HIV-associated TB.^1,3^ Several gaps need to be addressed to meet the End TB Strategy 2030 milestones and Kenya's Vision 2030 targets of reducing TB incidence by 80% and TB deaths by 90% compared to 2015.^2,4^

Pre-treatment loss to follow-up (PTLFU), when people diagnosed with TB are lost to care before starting TB treatment, is a major contributor to patient losses in the cascade of care.^5–7^ A systematic review of PTLFU by MacPherson et al. identified 23 studies from 14 countries, including eight African countries (not Kenya), and found that the proportion of people with TB experiencing PTLFU varied from 4% to 38%, with the highest proportion in Africa.^5^ In high TB burden settings, studies have reported high mortality associated with PTLFU and higher attrition rates from PTLFU than those observed during treatment.^5,6,8–10^ Most countries, including Kenya, do not include PTLFU as a routine indicator in their national TB programs; the extent of PTLFU is thus unknown.^5,11,12^

One 2014 national-level retrospective study found that 21% of 3,409 people with smear-positive TB were not recorded in the national TB surveillance system (TIBU) and may not have received treatment. The study also found that people with TB not reported in TIBU were more likely to be older (≥55 years), diagnosed from high-volume facilities and facilities in high TB burden regions in Kenya.^13^

To inform strategies to address PTLFU, we reviewed laboratory and treatment registers to determine the extent of PTLFU and associated patient factors in adults with pulmonary TB (PTB) in western Kenya.

## METHODS

### Study design and study site

This was a retrospective record review of laboratory and treatment registers at Jaramogi Oginga Odinga Teaching and Referral Hospital (JOOTRH), Kisumu, Kenya, among people with bacteriologically confirmed PTB.

JOOTRH in western Kenya, a high TB burden region,^13,14^ is a referral hospital for public, private and faith-based health facilities in over 10 counties, with a catchment population of approximately five million people, bed capacity of 710, offering outpatient and inpatient services.^13,15^ The main laboratory serves the JOOTRH TB clinic and 65 outside health facilities. Following the Kenyan TB guidelines, a sputum specimen is collected from individuals with presumptive TB and sent for Xpert^®^ MTB/RIF testing (Cepheid, Sunnyvale, CA, USA).^16^ Smear microscopy may be used to diagnose TB in people with presumptive TB whenever Xpert is unavailable, or cartridges are out of stock. Cultures are performed for people experiencing TB relapse before starting treatment and people whose sputum is persistently smear-positive.^16^ Test results are sent to the JOOTRH TB clinic and the respective referral facilities. In this area of western Kenya, Xpert testing is also available at one private and seven public health facilities.

**FIGURE 1.**
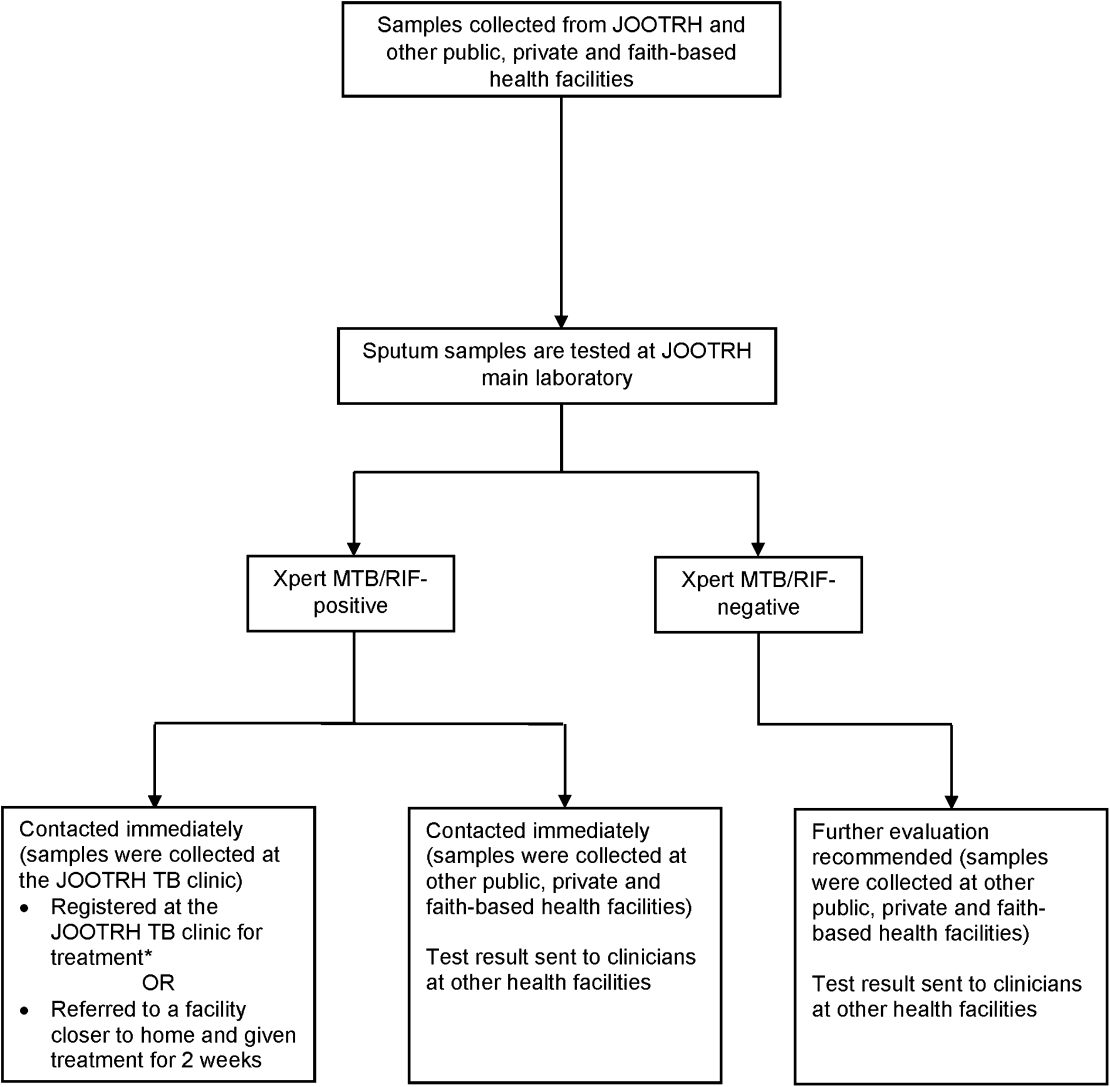
Diagram showing patient flow at JOOTRH TB programme. *Patient records for people with bacteriologically confirmed pulmonary TB who received care at the JOOTRH TB clinic were included in the study, see Figure 2. JOOTRH = Jaramogi Oginga Odinga Teaching and Referral Hospital.

JOOTRH staff promptly contacts individuals who test TB positive by telephone. Names, demographics and contact information of individuals who choose to receive care at the JOOTRH TB clinic start treatment are recorded in the TB treatment register. Those who prefer to receive care at another facility receive a 2-week treatment starter pack, are referred, and are contacted by telephone 1 month after receiving the test results by JOOTRH staff to establish their treatment location (Figure 1).

### Study sampling

We estimated a sample size of 249 would be sufficient to detect PTLFU. We utilised Fisher’s statistical formula with a precision of ±2.5% around the prevalence of PTLFU.^17^ Based on MacPherson et al., we used the weighted proportion of PTLFU from African studies to be 18%.^5^ We also factored in 10% to account for missing data.

**FIGURE 2.**
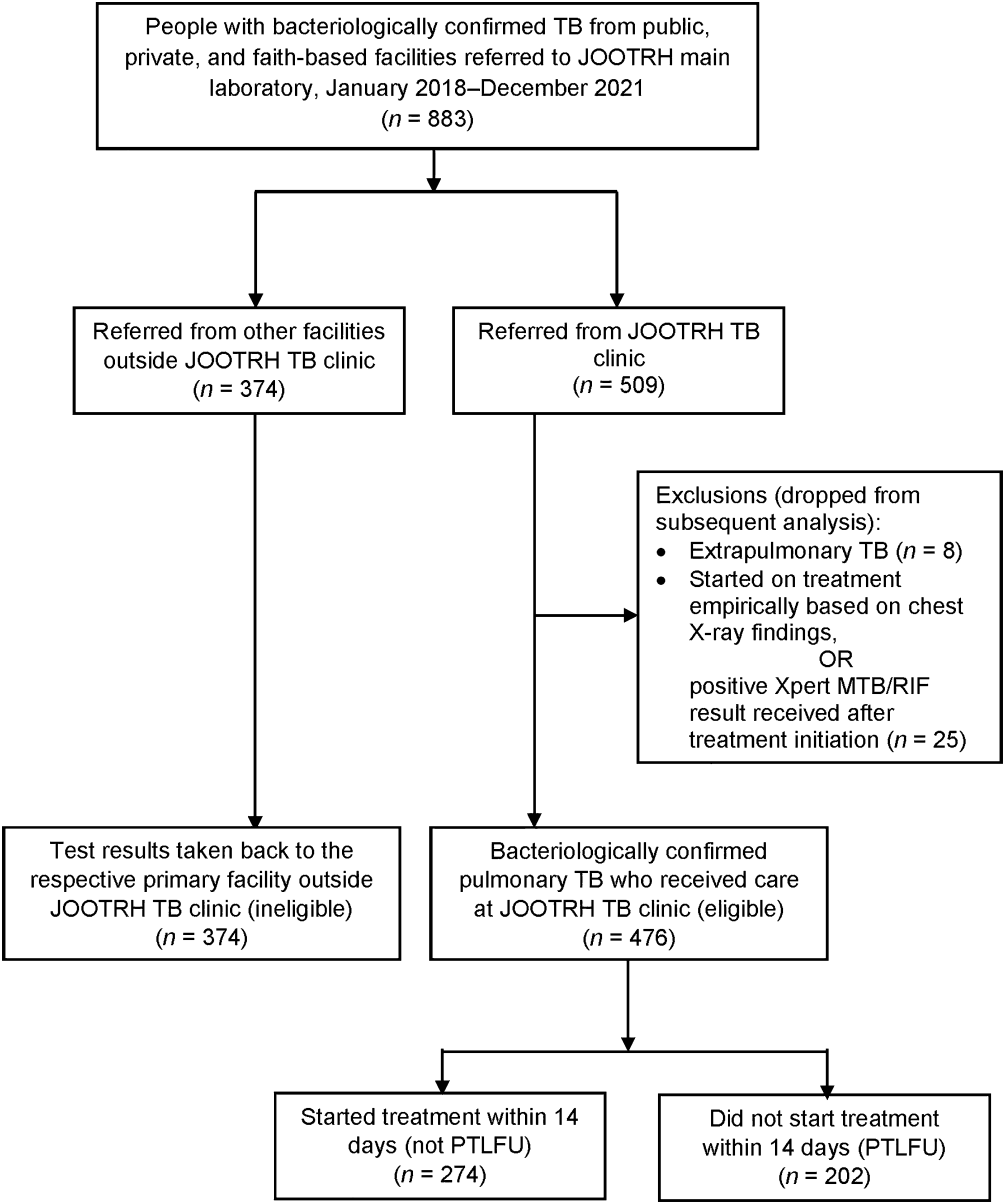
Flow chart showing selection of patient records of bacteriologically confirmed TB at JOOTRH, Kisumu, Kenya. JOOTRH = Jaramogi Oginga Odinga Teaching and Referral Hospital.

We used universal sampling and included all people with bacteriologically confirmed PTB, aged ≥18 years and evaluated at the JOOTRH TB clinic during the study period. People with bacteriologically confirmed PTB were excluded if their sputum samples were collected at outside health facilities and sent for testing at the JOOTRH main laboratory; however, their results were returned to the outside health facilities where TB care was provided. Patients with extrapulmonary TB and patients who started treatment empirically before receiving their Xpert test results were excluded (Figure 2).

### Pre-treatment loss to follow-up

PTLFU was defined based on previous studies as patients in a national TB care programme who received a diagnosis of TB based on at least one positive sputum smear or Xpert result but did not start TB treatment within 14 days. This included individuals who died before initiating treatment.^5,10,18^

We classified people as having PTLFU if they were 1) referred for testing from the JOOTRH TB clinic; 2) bacteriologically confirmed for PTB; and 3) if their names were recorded in the laboratory register, but not in the treatment register. People were classified as not having PTLFU if they were 1) referred for testing from the JOOTRH TB clinic; 2) bacteriologically confirmed for PTB; 3) if their names were recorded in the laboratory and treatment registers; and 4) if they started TB treatment within 14 days.

We traced all patients recorded in the treatment register using bio data (name, age and sex) from the laboratory register for at least 3 months from the date of PTB diagnosis to establish the date of treatment initiation. We did not track people who were considered to have experienced PTLFU.

### Study variables

Independent variables were sex, age, type of contact (telephone or physical address), place of residence (urban or rural), HIV status, history of TB treatment, linkage to TB treatment (started at the facility or referred) and the test used to verify the diagnosis of PTB.

The dependent variable was the number of days to initiation of treatment, defined as the difference between date of treatment initiation and the date of diagnosis (bacteriologically confirmed).

### Data collection

MM trained two research assistants on TB, study objectives, ethical principles and research instruments; piloted the data extraction form using 20 records, then modified and finalised the form; and collected data from the JOOTRH TB clinic laboratory and treatment registers in August and September 2022. Using the finalised form, we extracted laboratory serial number, date of laboratory examination, age, sex, contact information, place of residence, name of referral facility, HIV status, diagnostic test (Xpert testing or microscopy) and sample results from the laboratory register for all people who were bacteriologically confirmed with PTB from January 2018 to December 2021 (Supplementary Data 1). We used Research Electronic Data Capture (REDCap) tools hosted at Stellenbosch University (Tygerberg, South Africa) for efficient data management, including audit trails, automated export, seamless data downloads and interoperability with external sources.^19,20^

### Data management and analysis

The research assistants entered data into REDCap and MM checked daily for errors. Data were exported to STATA^®^ v17 (Stata, College Station, TX, USA), and descriptive statistics were calculated. For continuous data that were not normally distributed, range, median and interquartile ranges (IQRs) were reported. Age was collected as a continuous variable and later categorised into three age groups based on the Kenyan TB prevalence survey in 2015.^21^ We calculated the proportion of people experiencing PTLFU as the number of people who had initiated treatment more than 14 days from the date of diagnosis over the total number of people with bacteriologically confirmed PTB. We then used STATA’s ‘*proportion*’ command to obtain the 95% confidence intervals (CIs) of the proportion. We used the Pearson χ^2^ test to establish factors correlated with PTLFU.

Univariable logistic regression was used to obtain crude odds ratios (ORs) with 95% CIs and *P*-values, with *P* < 0.05 as the criterion for determining whether an observed difference was statistically significant. Multivariable logistic regression was performed to adjust for possible confounding factors. The likelihood ratio test was used to obtain the best-fit model. We included age, sex and HIV status in the final model as these are key risk factors for PTLFU.

### Ethical considerations and reporting

Ethical approval was obtained from the study site’s ethics and review committee JOOTRH, Kisumu, Kenya (IERC/JOOTRH/563/21); Kenya Medical Research Institute (KEMRI), Nairobi, Kenya (KEMRI/SERU/CGHR/392/4309), and Stellenbosch University, Tygerberg, South Africa (S21/04/066(PhD)). We removed personal identifiers before data analysis, stored data in a password-protected database and reported findings following the REporting of studies Conducted using Observational Routinely collected health Data (RECORD), an extension of the Strengthening the Reporting of Observational Studies in Epidemiology (STROBE) for routinely collected health data.^22^

## RESULTS

### General characteristics of the study population

Of 883 records of people diagnosed with TB between January 2018 to December 2021, 476 (53.9%) with bacteriologically confirmed PTB were eligible for the study (Figure 2). The mean age was 36.1 years (standard deviation [SD] ±12.9); 320 (67.2%) were male, 176 (37.0%) were HIV-positive and 27 (5.7%) had a history of anti-TB treatment. Of the total 476 participants, 459 (96.6%) had their contact information recorded, 235 (49.4%) had both a telephone number and physical address recorded and 438 (92.0%) had their place of residence recorded, with 299 (63.0%) being in an urban area. Regarding healthcare facility, 161 people (33.8%) started treatment at the study facility and 122 (25.6%) were referred to another facility for treatment. Information on referrals to other facilities for treatment was missing for 193 participants (40.6%). The median time to treatment initiation after diagnosis was 0 days (IQR 0–1, range 0 –377; Table 1).

**TABLE 1.** Demographic characteristics and clinical profile of adults with pulmonary TB at JOOTRH, Kisumu, Kenya.

Variable	(*n* = 476)
	*n* (%)
Sex	
Male	320 (67.2)
Female	155 (32.6)
Missing	1 (0.2)
Age group, years	
15–34	244 (51.3)
35–54	182 (38.2)
≥55	48 (10.1)
Missing	2 (0.4)
Contact information available	
Yes	459 (96.6)
No	16 (3.4)
Missing	1 (0.2)
Type of contact information	
None	17 (3.6)
Physical address	198 (41.6)
Telephone number	26 (5.5)
Telephone number and physical address	235 (49.4)
Place of residence recorded	
No	38 (8.0)
Yes	438 (92.0)
Place of residence	
Urban	299 (63.0)
Rural	139 (29.2)
Missing	38 (7.8)
HIV status	
Negative	271 (56.9)
Positive	176 (37.0)
Unknown	29 (6.1)
Diagnostic test	
Smear microscopy	1 (0.2)
Xpert MTB/RIF	475 (99.8)
Patient referred to another facility for treatment	
No	161 (33.8)
Yes	122 (25.6)
Unknown	193 (40.6)
History of anti-TB treatment	
No	257 (54.1)
Yes	27 (5.7)
Unknown	192 (40.2)

JOOTRH **=** Jaramogi Oginga Odinga Teaching and Referral Hospital.

### Pre-treatment loss to follow-up

Overall, 202 people with bacteriologically confirmed PTB (42.4%, 95% CI 38.1–46.9) experienced PTLFU, nine of whom (4.5%) started on treatment after 14 days from the date of diagnosis (Table 2 and Supplementary Table S1). Using univariable logistic regression, variables found to be associated with PTLFU included contact provided, type of contact and place of residence recorded. On multivariable analysis, the odds of experiencing PTLFU with only a physical address recorded was 34 times compared to when both physical address and telephone contact were recorded (adjusted OR [aOR] 34.2, 95% CI 18.7–62.5; *P* = 0.000). The odds of experiencing PTLFU with only a recorded telephone number was 22 times compared to when both physical address and telephone contact were recorded (aOR 22.3, 95% CI 3.5–141.1, *P* = 0.001). Patients aged ≥55 years were about three times more likely to experience PTLFU than those aged 15–34 years (aOR 2.6, 95% CI 1.0–6.7; *P* = 0.046). Sex, HIV status, place of residence recorded and history of anti-TB treatment were not found to be associated with PTLFU after controlling for confounders (Table 3).

**TABLE 2.** Pre-treatment loss to follow-up in adults with pulmonary TB at JOOTRH, Kisumu, Kenya.

Treatment initiation	*n*	% (95%CI)
≤14 days after diagnosis	274	57.6 (53.1–61.9)
>14 days after diagnosis*	202	42.4 (38.1–46.9)
Total	476	100.0

* Meets criterion for pre-treatment loss for follow-up. Nine people with TB started treatment after 14 days; 193 people were listed in the laboratory register, but not in the treatment register.

JOOTRH = Jaramogi Oginga Odinga Teaching and Referral Hospital; CI = confidence interval.

**TABLE 3.** Factors associated with pre-treatment loss to follow-up in patients with pulmonary TB at JOOTRH (Kisumu, Kenya) in multivariable logistic regression (final number of observations = 444).

	Univariable		Multivariable	
Variable	OR (95% CI)	*P*-value	aOR (95%CI)	*P*-value
Sex				
Male	1.0		1.0	
Female	0.9 (0.4–1.4)	0.753	0.8 (0.5–1.5)	0.509
Age, years				
15–34	1.0		1.0	
35–54	1.0 (0.7–1.4)	0.856	1.0 (0.6–1.7)	0.930
≥55	1.9 (1.0–3.5)	0.053	2.6 (1.0–6.7)	0.046
Contact provided*				
Yes	1.0			
No	6.2 (1.7–22.0)	0.005		
Type of contact				
Telephone number and physical address	1.0		1.0	
None	39.2 (11.6–132.6)	0.000	3.3 (0.2–69.4)	0.438
Physical address	35.7 (20.0–63.6)	0.000	34.2 (18.7–62.5)	0.000
Telephone number	92.4 (25.3–337.7)	0.000	22.3 (3.5–141.1)	0.001
Residence recorded				
Yes	1.0		1.0	
No	13.7 (4.8–39.2)	0.000	9.0 (0.6–126.8)	0.103
Place of residence*				
Urban	1.0			
Rural	0.9 (0.6–1.4)	0.781		
HIV status				
Negative	1.0		1.0	
Positive	0.9 (0.6–1.4)	0.674	1.0 (0.5–1.7)	0.857
History of anti-TB treatment*				
No	1.0			
Yes	1.1 (0.1–8.7)	0.957		

* Contact provided, place of residence and history of anti-TB treatment variables; aORs could not be estimated in the multivariable model owing to missing data.

JOOTRH = Jaramogi Oginga Odinga Teaching and Referral Hospital; OR = odds ratio; CI = confidence interval; aOR = adjusted OR.

## DISCUSSION

Notwithstanding the availability of free anti-TB treatment in Kenya, we found PTLFU to be higher (42.4%) in western Kenya than in other studies.^6,7^ Systematic reviews by MacPherson et al. (14 countries) and Subbaraman et al. (India) found respectively 4–38% and 16% of people with TB experienced PTLFU.^5,6^

Previous studies have utilised different cut-offs to define PTLFU, ranging from 2 days to 3 months, making it challenging to compare results across settings.^10,11,18,23–25^ The higher proportion of PTLFU in our study could be due to the longer study period, 4 years compared to up to 1 year in other studies.^10,11,24,25^ Furthermore, our study was conducted in an urban high-volume facility.^12,18^ In Cameroon, a study found that TB diagnostic and treatment units (DTUs) located in an urban area and travelling more than 30 km to the DTU were factors contributing to PTLFU.^11^ As 37% of study participants were HIV-positive, there is a possibility that some people with TB died before starting treatment.

We found that people who provided only a physical address or telephone number were more likely to experience PTLFU than those who provided both. Of interest, people who did not provide any contact were less likely to experience PTLFU, which intuitively appears illogical and differs from what other studies have reported.^18,26^ As reported in studies from South Africa and India,^27,28^ this might be linked to people providing incorrect information to prevent being tracked and identified as having TB and concerns about the association of TB with HIV.^27–29^ Similar to studies in India,^18,28,30^ older people affected by TB (≥55 years) were more likely to experience PTLFU than people aged 15–34 years. Older people may have difficulty returning to the health facility without assistance.

A systematic review by MacPherson et al. and a review by Subbaraman et al. identified key patient and health system factors associated with PTLFU.^5,31^ Patient-related factors included older age, male sex, TB history, urban location and weakness due to advanced disease.^5,31^ Health system-related factors included difficulty in navigating between health facilities, frequent visits to the health facility, delays in receiving test results and geographical location of the TB laboratory.^5,31^ Similar to the aforementioned studies, we identified older age as a factor associated with PTLFU.^5,31^ In contrast with the review by Subbaraman et al., we did not find anti-TB history to be associated with PTLFU.^31^

Studies tracking people affected by TB have found higher mortality in people experiencing PTLFU than those starting treatment.^8–10^ This suggests that TB programmes may be underestimating TB mortality by not accounting for people experiencing PTLFU.^5^ Adding PTLFU as a quarterly indicator would help to report and may contribute to improved patient outcomes and attainment of End TB Strategy goals.^2^

Our findings contribute to the body of evidence on PTLFU in Kenya. We used routinely collected data revealing the on-the-ground reality of the TB programme in western Kenya. We piloted our data extraction form, used trained staff for data collection and reported our findings according to RECORD guidelines.^22^ This study had some limitations. We reviewed TB programme records to determine PTLFU and did not trace patients whose names were recorded in the laboratory register but not the treatment register to ascertain whether they had started treatment within 14 days at another facility. The substantial amount of missing data (40.6%) regarding whether participants were referred to other facilities limits the strength of our conclusions. Missing and incomplete information may have introduced selection bias and led to an overestimation or underestimation of PTLFU. Although the study facility had a wide catchment area, it might not be representative of other parts of the country. We were unable to collect certain key patient information such as level of education, employment status, level of income, comorbidities and distance to the study facility from the patient’s residence, which are important risk factors for PTLFU.^28,32,33^

## CONCLUSION

In western Kenya, adults with PTB experience a high proportion of PTLFU, with limited contact details and older age being risk factors. Strengthening systems for documenting patient information and actively monitoring PTLFU are crucial to address PTLFU. To aid patient tracking, healthcare facilities ought to employ personnel who are solely dedicated to recording detailed contact information of patients and explore the feasibility of electronically linking laboratory and treatment registers. A qualitative study of patient perspectives on PTLFU should be undertaken to complement these findings. Prospective studies on PTLFU in other parts of Kenya are also needed.

## Supplementary Material


