# Supplementary material for: Pre-treatment loss to follow-up in adults with pulmonary TB in Kenya
Source: Public Health Action. 2024 Mar 1;14(1):34–9. doi: 10.5588/pha.23.0059 (PMC11122711; doi:10.5588/pha.23.0059)
Supplement: Supplementary file 1 [file iutld_pha_23.0059_supplementarydata1.pdf]

http://dx.doi.org/10.5588/pha.23.0059

SUPPLEMENTARY DATA

Pre-treatment loss to follow-up in adults with pulmonary TB in Kenya

Supplementary Data 1: Data collection tool

Instructions

(i) Data to be collected from the laboratory and treatment register at the Tuberculosis clinic

(ii) Please indicate with a tick (√) in the boxes and table below the appropriate response.

(iii) If the information is missing indicate (missing).

A. BIODATA

Record serial number

____

Research Assistants initials

____

Today's Date (Date when data is being entered)

____

Age in years

____

Enrolment date (Date enrolled at the clinic)

____

Contact Information

____

	Yes	No	Unknown/not recorded
**Gender**			
Male			
Female			
Unknown			
**Level of education**			
None			
Primary			
Secondary			
Tertiary(college/University)			
Unknown			
**Employment status**			
Employed			
Unknown			
**Marital status**			
Married			
Not Married			
Unknown			
**Place of residence**			
Rural			
Semi-urban			
Urban			
Unknown			

B. PROCESS AND OUTCOME TOOL

**No.**	**Question**	**Yes**	**No**	**Notes/Date**
**Individualised assessment for the patient**				
1	Has the patient been diagnosed with TB?			
2	Was there a laboratory confirmation?			
3	Which type of TB<br>• Pulmonary TB			
	• Extra-pulmonary TB			
4	Which test was used?<br>• Xpert MTB/RIF			
	• Smear microscopy			
	• Chest X-ray			
5.	HIV status?			

	Positive			
	Negative			
	Unknown			
6.	Was the patient referred for treatment?			
**Initiating treatment**				
7	Was patient history taken?			
	Was a physical examination done?			
8	Has the patient been treated for TB before?			
9.	Has the patient been started on treatment?			
**Additional clinical conditions**				
	COVID-19			
	Malaria			
	HIV/AIDS			
	Diabetes			
	Asthma			
	COPD			
	Hypertension			
	Others (Specify)			

Table 1: Outcomes

**DATE**	Started treatment at the study hospital	Referred to a local facility and started treatment at the facility	No record of the patient returning for results	Received results but did not return to the clinic for treatment	Died

Supplementary Table S1:Comparison of characteristics of those who experience PTLFU and those who did not experience PTLFU in patients with pulmonary tuberculosis at JOOTRH

Predictor variable		PTLFU (n=202)	NO PTLFU (n=274)	p-value
Sex (n=476)	Male	137 (68.2%)	183 (66.8) %)	0.753
	Female	64 (31.8%)	91 (33.2%)	
	Unknown	1 (0.5%)	0 (0.0%)	
Age in years (n=474)	15-34	100 (50.0%)	144 (52.6%)	0.113
	35-54	73 (36.5%)	109 (39.8%)	
	≥55	27 (13.5%)	21 (7.7%)	
Contact provided (n=475)	No	13 (6.4%)	3 (1.1%)	0.001
	Yes	189 (93.6%)	270 (98.9%)	
Type of contact (n=476)	None	13 (6.4%)	4 (1.5%)	0.000
	Physical address	148 (73.3%)	50 (18.3%)	
	Telephone number	23 (11.4%)	3 (1.1%)	
	Telephone number and physical address	18 (8.9%)	217 (79.2%)	
Residence recorded (n=476)	No	34 (16.8%)	4 (1.5%)	0.000
	Yes	168 (83.2%)	270 (98.5%)	
Place of residence (n=438)	Rural	52 (31.0%)	87 (32.2%)	0.781
	Urban	116 (69.1%)	183 (67.8%)	
HIV status (n=476)	Negative	107 (53.0%)	164 (59.9%)	0.000
	Positive	66 (32.7%)	110 (40.2%)	
	Unknown	29 (14.4%)	0 (0.0%)	
History of antituberculosis treatment (n=476)	No	9 (4.5%)	248 (90.5%)	0.000
	Yes	1 (0.5%)	26 (9.5%)	
	Unknown	192 (95.1%)	0 (0.0%)	
